# Evaluation of an E-Learning Training Program to Support Implementation of a Group-Based, Theory-Driven, Self-Management Intervention For Osteoarthritis and Low-Back Pain: Pre-Post Study

**DOI:** 10.2196/11123

**Published:** 2019-03-07

**Authors:** Deirdre A Hurley, Alison Keogh, Danielle Mc Ardle, Amanda M Hall, Helen Richmond, Suzanne Guerin, Tara Magdalinski, James Matthews

**Affiliations:** 1 School of Public Health, Physiotherapy and Sports Science University College Dublin Dublin Ireland; 2 Faculty of Medicine Memorial University St Johns, NL Canada; 3 Centre for Rehabilitation Research in Oxford (RRiO) Nuffield Department of Orthopaedics, Rheumatology and Musculoskeletal Sciences University of Oxford Oxford United Kingdom; 4 School of Psychology University College Dublin Dublin Ireland; 5 Faculty of Health, Arts and Design Swinburne University of Technology Melbourne Australia

**Keywords:** technology-enhanced learning, evaluation, e-learning, digital learning, program evaluation, effectiveness, physiotherapy, implementation, osteoarthritis, low-back pain

## Abstract

**Background:**

By adaptation of the face-to-face physiotherapist-training program previously used in the Self-management of Osteoarthritis and Low back pain through Activity and Skills (SOLAS) feasibility trial, an asynchronous, interactive, Web-based, e-learning training program (E-SOLAS) underpinned by behavior and learning theories was developed.

**Objective:**

This study investigated the effect of the E-SOLAS training program on relevant outcomes of effective training and implementation.

**Methods:**

Thirteen physiotherapists from across Ireland were trained via E-SOLAS by using mixed methods, and seven physiotherapists progressed to implementation of the 6-week group-based SOLAS intervention. The effectiveness of E-SOLAS was evaluated using the Kirkpatrick model at the levels of reaction (physiotherapist engagement and satisfaction with E-SOLAS training methods and content), learning (pre- to posttraining changes in physiotherapists’ confidence and knowledge in delivering SOLAS content and self-determination theory-based communication strategies, administered via a SurveyMonkey questionnaire), and behavior (fidelity to delivery of SOLAS content using physiotherapist-completed weekly checklists). During implementation, five physiotherapists audio recorded delivery of one class, and the communication between physiotherapists and clients was assessed using the Health Care Climate Questionnaire (HCCQ), the Controlling Coach Behaviour Scale (CCBS), and an intervention-specific measure (ISM; 7-point Likert scale). A range of implementation outcomes were evaluated during training and delivery (ie, acceptability, appropriateness, feasibility, fidelity, and sustainability of E-SOLAS) using a posttraining feedback questionnaire and individual semistructured telephone interviews.

**Results:**

With regard to their reaction, physiotherapists (n=13) were very satisfied with E-SOLAS posttraining (median 5.0; interquartile range 1.0; min-max 4.0-5.0) and completed training within 3-4 weeks. With regard to learning, there were significant increases in physiotherapists’ confidence and knowledge in delivery of all SOLAS intervention components (*P*<.05). Physiotherapists’ confidence in 7 of 10 self-determination theory-based communication strategies increased (*P*<.05), whereas physiotherapists’ knowledge of self-determination theory-based strategies remained high posttraining (*P*>.05). In terms of behavior, physiotherapists delivered SOLAS in a needs supportive manner (HCCQ: median 5.2, interquartile range 1.3, min-max 3.7-5.8; CCBS: median 6.6, interquartile range 1.0, min-max 5.6-7.0; ISM: median 4.5, interquartile range 1.2, min-max 2.8-4.8). Fidelity scores were high for SOLAS content delivery (total %mean fidelity score 93.5%; SD 4.9%). The posttraining questionnaire and postdelivery qualitative interviews showed that physiotherapists found E-SOLAS acceptable, appropriate, feasible, and sustainable within primary care services to support the implementation of the SOLAS intervention.

**Conclusions:**

This study provides preliminary evidence of the effectiveness, acceptability, and feasibility of an e-learning program to train physiotherapists to deliver a group-based self-management complex intervention in primary care settings, which is equivalent to face-to-face training outcomes and would support inclusion of physiotherapists in a definitive trial of SOLAS.

## Introduction

International clinical guidelines for osteoarthritis and low-back pain endorse self-management, exercise, and physical activity as key components of health care interventions [1-4], but the evidence for their effectiveness is weak and of low quality [5-7]. The Self-management of Osteoarthritis and Low back pain through Activity and Skills (SOLAS) intervention is an evidence-supported group treatment approach developed through intervention mapping [8], which is a logical six-step process for the development and evaluation of theory-driven and evidence-based interventions that takes into account stakeholder needs and the practicalities of implementation [9]. SOLAS was evaluated for its acceptability and preliminary effects in comparison with individual physiotherapy in a feasibility trial (trial registration: ISRCTN49875385) set in Dublin, Ireland, between September 2014 and June 2016 [10]. Intervention physiotherapists who participated in the trial were trained using brief interactive lectures, videos, role play, and practical skills to deliver the SOLAS intervention using communication skills underpinned by self-determination theory. This theory proposes that people have basic psychological needs for autonomy, competence, and relatedness, which if met, for example, by the needs supportive communication style of a health care practitioner (HCP), will increase an individual’s autonomous motivation and engagement in health behaviors such as self-management [11]. The Medical Research Council guidelines recommend that complex behavior-change programs train their intervention deliverers to ensure implementation with high fidelity [12]. Hence, the Kirkpatrick model was used to evaluate training at the levels of Reaction, Learning, and Behavior [13], which showed that physiotherapists were satisfied with face-to-face training and their confidence in the self-determination theory-based communication strategies. Knowledge of the intervention content significantly increased, and the physiotherapists delivered SOLAS in a needs supportive manner with high fidelity to the intervention content [14,15]. Upscaling to a definitive national trial would render the face-to-face training impractical for physiotherapists due to significant time, travel, and costs constraints [16]. Therefore, we subsequently developed an asynchronous, interactive, Web-based, e-learning training program for SOLAS (E-SOLAS) to prepare physiotherapists to deliver the SOLAS intervention. If successful, the program would reduce the time needed to move to a definitive trial. Furthermore, E-SOLAS has the potential to increase the competencies of physiotherapists with regard to self-management behavior-change skills in line with the shared strategic priority of Ireland’s public health service and higher education institutions to train and prepare future health care graduates with the skills necessary to support lifestyle behavior change in their patients [17,18], making the intervention more accessible to physiotherapists for long-term sustainability.

Despite the increased availability of e-learning training for HCPs internationally, there is limited formal evaluation of such training programs. Current evidence, which predominantly involves undergraduate HCP students [19,20], suggests that e-learning shows similar effectiveness to traditional methods for knowledge acquisition [21] and user satisfaction [22], but further research regarding the effectiveness of e-learning on HCP behavior change and the translation of learning to clinical practice has been advocated [23]. Hence, we evaluated E-SOLAS in the same way as our face-to-face training. In addition, a range of World Health Organization–recommended implementation outcomes were included for evaluation, including the acceptability, appropriateness, feasibility, fidelity, and sustainability of E-SOLAS, in a range of primary care physiotherapist settings across Ireland [24,25] in order to understand the contextual elements of e-learning [16].

The study objectives were to evaluate the effect of the E-SOLAS training program on physiotherapists’ reaction, learning, and delivery of the SOLAS intervention as intended and to assess the acceptability, appropriateness, feasibility, fidelity, and sustainability of E-SOLAS to aid the implementation of the SOLAS intervention in primary care settings.

## Methods

### Study Design and Research Ethics

This was a single-group, pre-post study. Ethical approval was granted by the UCD Human Subject (Sciences) Ethics Committee in two phases: in Phase 1, for the E-SOLAS training program (September 30, 2016; LS-E-16-121-Hurley) and in Phase 2, for implementation of the SOLAS intervention (December 21, 2016; LS-16-97-Hurley). The study was also approved for Phase 1 (November 17, 2016) and Phase 2 (January 17, 2017) by the Health Service Executive Primary Care Research Committee.

### Participants and Procedure

Physiotherapy managers from 10 primary care areas across Ireland who had not participated in the SOLAS feasibility trial were sent a study information leaflet for screening based on their service facilities and staffing capabilities. Seven physiotherapy managers fulfilled the criteria for inclusion, provided letters of support, and nominated two staff members to undertake E-SOLAS training. Nominated physiotherapist staff were sent the study information leaflet and consent form. Consenting participants were required to possess a device that could connect to the internet and were given password-protected access to the social learning platform Curatr [26] that hosted the E-SOLAS training program. Participants were encouraged to complete the training over a 4-week period by working at their own pace and at times that were convenient for them. During training, they had access to ongoing technical support from the research team and were requested to keep a log of the time spent on each aspect of training.

At the end of the training period, participants were invited to set up and deliver the SOLAS intervention according to the treatment protocol [10] in each of their primary care areas. Physiotherapists had ongoing access to E-SOLAS during implementation and were provided with any additional intervention materials required to deliver the intervention by the research team (ie, intervention PowerPoint [Microsoft Corp, Redmond, WA] slide deck on a universal serial bus, pedometers, and relaxation CDs for each client). Following completion of the 6-week delivery phase, each physiotherapist was invited to participate in an individual semistructured telephone interview to explore their views of E-SOLAS as a tool to support implementation of the intervention.

### E-SOLAS Training Program

E-SOLAS is a Web-based e-learning training program designed to train physiotherapists to deliver a group-based education and exercise intervention for patients with osteoarthritis and chronic low-back pain. The content is based on the face-to-face training program developed for the SOLAS feasibility trial [10,15]. The E-SOLAS program is hosted on Curatr [26], an online social learning platform that creates a collaborative learning environment and uses gamification principles. The development process for the E-SOLAS program is outlined in Multimedia Appendix 1 [27-31].

### E-SOLAS Program Structure and Content

**Figure 1:**
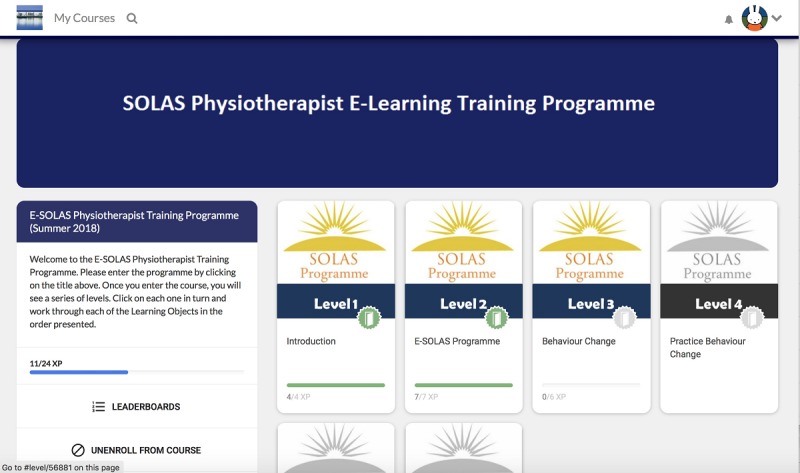
E-SOLAS home page screenshot. E-SOLAS: E-learning training program for Self-management of Osteoarthritis and Low back pain through Activity and Skills.

The E-SOLAS program contained six hierarchical linear levels, whereby the user was required to finish each level before progressing to the next level (Multimedia Appendix 2, Figure 1).

**Figure 2:**
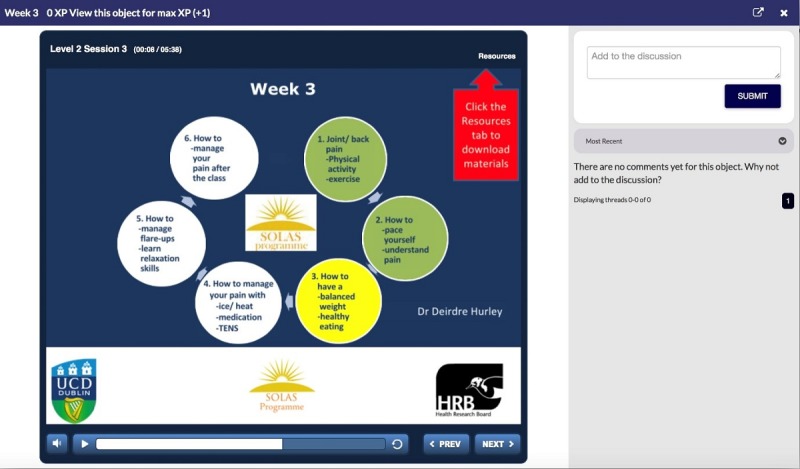
E-SOLAS program content screenshot. E-SOLAS: E-learning training program for Self-management of Osteoarthritis and Low back pain through Activity and Skills.

**Figure 3:**
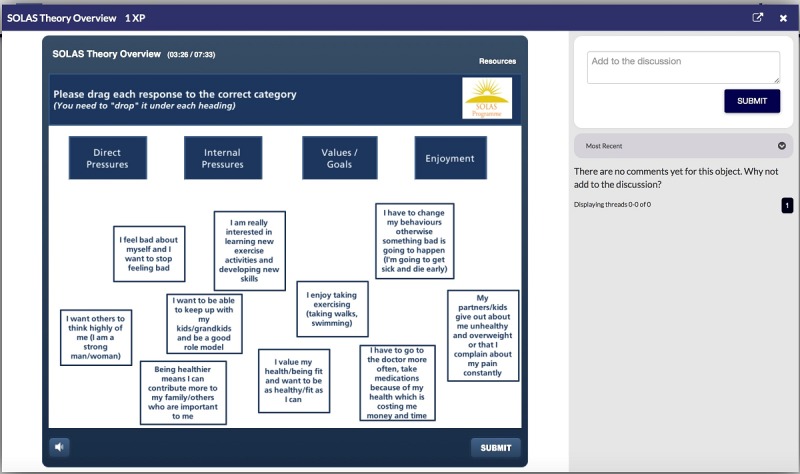
E-SOLAS theory screenshot. E-SOLAS: E-learning training program for Self-management of Osteoarthritis and Low back pain through Activity and Skills.

Briefly, the program begins in Level 1 with an overview of the training program and the SOLAS intervention. Level 2 describes the education content for each week of the SOLAS intervention (eg, the key learning points and the materials required; Figure 2). At Level 3, the self-determination theory-based communication strategies that physiotherapists use as part of the intervention are introduced (Figure 3), and in Level 4, they are given the opportunity to role play these strategies. Level 5 highlights the exercises and their mode of delivery, and finally, Level 6 concludes the program by highlighting the next steps for intervention delivery.

**Figure 4:**
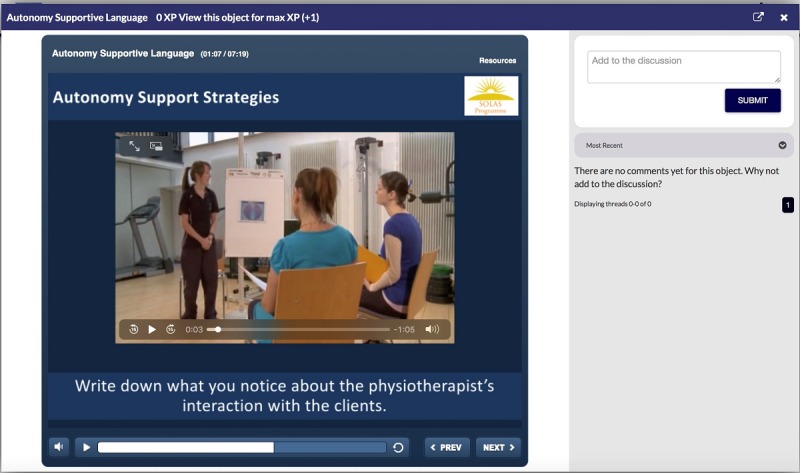
E-SOLAS video-based program activity screenshot. E-SOLAS: E-learning training program for Self-management of Osteoarthritis and Low back pain through Activity and Skills.

Throughout each level, there are lectures with voice-overs, video examples of good and poor practice (Figure 4), videos from the research team, and a peer role model explaining certain elements of the intervention; short “in level” activities and self-reflection opportunities; and end-of-level “gate” assessments with varying levels and modes of feedback depending on the activity.

### Outcome Measures

The effectiveness of the E-SOLAS training program was assessed using the Kirkpatrick model of evaluation at the levels of reaction, learning, and behavior [13]. Furthermore, a range of implementation outcomes were evaluated during the training and intervention-delivery phases. The measurement tools used to assess learning and implementation outcomes are described in detail in Multimedia Appendix 3 and briefly outlined below.

#### Training Outcomes

##### Reaction

To measure physiotherapists’ reaction to E-SOLAS, a researcher-devised feedback measure was developed by adapting the face-to-face training feedback measure [15] and incorporating factors related to the evaluation of technology enhanced learning [25]. It was administered following posttraining assessment via SurveyMonkey and included items related to participant satisfaction, engagement, accessibility, and quality of E-SOLAS as well as several implementation outcomes as detailed below (Multimedia Appendix 4). Physiotherapists’ engagement with E-SOLAS was further evaluated using Curatr analytics [26] and a self-reported activity log completed by each physiotherapist during training.

##### Learning

Learning was assessed by evaluating physiotherapists’ perceptions of self-reported knowledge and confidence pre- and posttraining (Multimedia Appendix 5) and their use of skills during training by using a range of measures.

##### Behavior

Physiotherapists’ behavior was assessed during delivery of the SOLAS intervention to evaluate fidelity to the intervention content and self-determination theory-based communication strategies using previously validated checklists [14] and audio recordings [15]. In line with fidelity guidelines [32], each audio recording was coded by one blinded expert rater (AK) to assess physiotherapists’ communication style [33], and three audio recordings were coded by a second expert rater (JM). The Health Care Climate Questionnaire (HCCQ) [34] was the primary measure to assess provider delivery of the self-determination theory-based communication style, with an adapted version of the Controlling Coach Behaviour Scale (CCBS) [35] and an intervention-specific SOLAS scale used as secondary measures [15].

#### Implementation Outcomes

Implementation outcomes were measured through specific items on the feedback measure related to the acceptability, appropriateness, feasibility, and sustainability of E-SOLAS (Multimedia Appendix 4) and an individual semistructured telephone interview of the physiotherapists conducted by an experienced qualitative researcher (SG) within 2 weeks of completing group class delivery. A topic guide was developed for the participant interviews with specific questions and probes related to their views of E-SOLAS as a model of training in order to support physiotherapists in delivering the SOLAS intervention in primary care settings. All interviews were audio recorded.

### Data Analysis

Data from all outcome measures were analyzed using Excel (version 14.2.3, Microsoft Corp) and a statistical software package (SPSS Statistics, version 20, IBM Corp, Armonk, NY) following checks for errors in data entry.

#### Training Outcomes

##### Reaction

In order to assess physiotherapists’ views of their satisfaction, accessibility, and quality of the E-SOLAS program and their engagement with the e-learning training, descriptive statistics were used to analyze quantitative data, and thematic analysis was used to analyze free-text answers.

##### Learning

###### Analytical Methods

Descriptive statistics were used to calculate scores pre- and posttraining for overall confidence in delivering SOLAS content, the specific SOLAS intervention components, and the use of each self-determination theory-based communication strategy. Differences between pre- and posttraining were calculated using the Wilcoxon signed-rank tests and adjusted for multiplicity using Bonferroni corrections (0.05/n tests).

###### Knowledge

Descriptive statistics were used to calculate the level of SOLAS intervention knowledge, and pre- and posttraining differences were calculated using the Wilcoxon signed-rank tests. Following discussion between raters, there was excellent agreement (100%) in the coding of physiotherapists’ narrative case studies. The number of self-determination theory-based communication strategies used by each physiotherapists and the percentage of physiotherapists who used each strategy was calculated, with differences in the rate of use of all strategies and each strategy pre- and posttraining determined using McNemar tests. All results were adjusted for multiplicity using Bonferroni corrections.

###### Skills

Each role-play audio recording was rated for the use of self-determination theory-based communications strategies on a 7-point Likert scale ranging from “1 - not at all well” to “7 - very well,” with values at or above the mid-point of the Likert scale (4/7) defined as demonstrating skills that could be considered acceptable in terms of competence [15].

##### Behavior

The mean fidelity levels to SOLAS intervention content and fidelity levels according to physiotherapist, site, session, and session category were obtained by calculating total actual scores as a percentage of the total possible score using checklists. Fidelity of duration was established by calculating the difference between the actual and the intended session durations using a one-sample Wilcoxon test. Levels of fidelity were interpreted as previously reported in the literature [36]. A review of the raters’ scores for the audio recordings of physiotherapists’ delivery of SOLAS session 4 demonstrated excellent agreement (90%). To establish physiotherapists’ competence in the self-determination theory-based communication style, a median result for each of the three outcome measures was calculated separately. For the SOLAS scale, a median score per construct subsection (eg, autonomy), subcomponent strategy (eg, positive feedback), and class component (eg, education) was also calculated.

#### Implementation Outcomes

Descriptive statistics were used to analyze quantitative data related to physiotherapists’ views of the acceptability, appropriateness, feasibility, and sustainability of E-SOLAS. Qualitative data from the physiotherapists’ interviews were transcribed verbatim and analyzed using inductive thematic analysis [37]. A coding frame was developed from a review of provisional themes, which were then reexamined and refined (DMcA). The reliability of the identified themes was established by a second researcher (DAH) who independently coded a random sample of 25% of each dataset using the coding frame, with 70% agreement taken as the minimum cut-off rate [10]. The level of agreement between raters was 85%.

## Results

### Principal Findings

**Figure 5:**
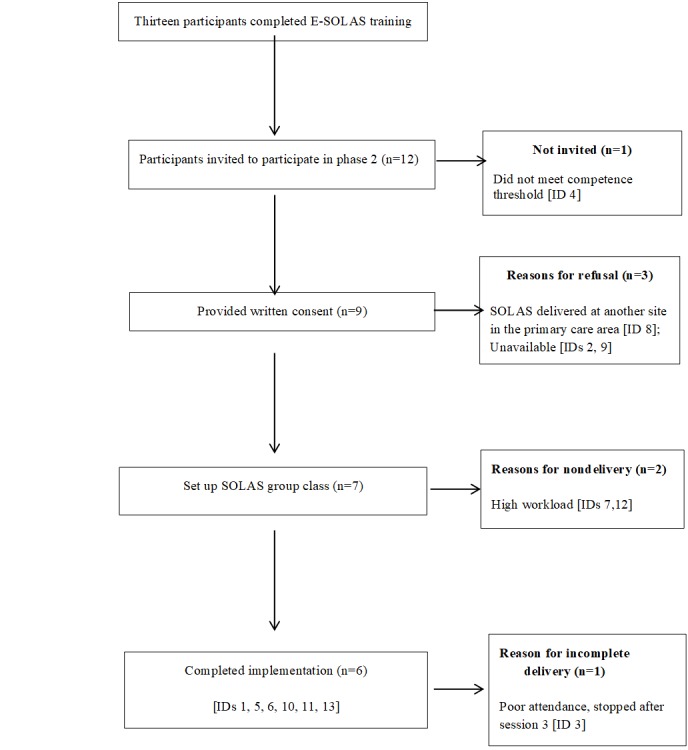
Participant flow through the study. E-SOLAS: E-learning training program for Self-management of Osteoarthritis and Low back pain through Activity and Skills; ID: participant identification number.

Thirteen physiotherapists from seven primary care areas completed the E-SOLAS training, of which 12 were invited to participate in the implementation study (ie, delivery of the SOLAS intervention). Nine physiotherapists consented to participate, and seven progressed to deliver SOLAS. The profile of physiotherapists in each study phase is provided in Table 1, and the flow of participants through Phase 2 is outlined in Figure 5. The training and delivery groups were comparable for the majority of descriptive variables, apart from the median years qualified, which was lower in the delivery group.

### Training Outcomes

#### Reaction

Physiotherapists (n=13) were very satisfied with E-SOLAS training posttraining and found it enjoyable and engaging, with all participants completing the program within the 4 weeks available (Multimedia Appendix 4). Physiotherapists reported that they spent a mean of 9.1 (SD 3.3) hours (min-max 4.1-16.1) over 16.3 (SD 6.0) days to complete E-SOLAS, which was not statistically different from the duration of training recorded by Curatr analytics (mean difference –1.69; SD 4.37; *t*=–1.397; *df*=12; *P*=.19; Multimedia Appendix 6). All physiotherapists successfully completed all-level gate assessments and the required three uploads and made at least one online posting to the group discussion. The majority of physiotherapists reported completing E-SOLAS outside work hours and spent 1-2 hours at any one time on training. The most commonly cited positive features of E-SOLAS were the range of brief video clips (46.2%; n=6) and focus on communication skills and client motivation (23.1%; n=3). Nine of the 13 participants experienced some difficulties during training; the most common difficulty was related to accessing online materials (46.2%, n=6), completing gate assessments (38.5%, n=5), and computer access at work (30.8%, n=4). Although the median ratings for working independently and not having access to other therapists were very positive, four physiotherapists required support from the University College Dublin team during training for accessing resources (n=3), logging into E-SOLAS via work email (n=2), or uploading audio files (n=1). Nonetheless, the majority of physiotherapists highly rated the quality of the training program and format.

**Table 1:** Baseline characteristics of physiotherapists.

Demographic characteristics			Training group (n=13)	Delivery group (n=7)
**Gender, n (%)**				
	Male		2.0 (15.4)	1.0 (14.3)
	Female		11.0 (84.6)	6.0 (85.7)
**Age (years), n (%)**				
	26-35		2.0 (15.4)	2.0 (28.6)
	36-45		4.0 (30.8)	2.0 (28.6)
	46-55		3.0 (23.0)	1.0 (14.2)
	56-65		4.0 (30.8)	2.0 (28.6)
**Number of physiotherapists in the primary care area (ID^a^)**				
	Area 1		2.0 (4,7)	0
	Area 2		1.0 (2)	0
	Area 3		2.0 (6, 8)	1.0 (6; Site 2)^b^
	Area 4		2.0 (5, 13)	2.0 (5 and 13; Site 4)^c^
	Area 5		2.0 (1, 3)	2.0 (1, Site 1; 3, Site 6)^d^
	Area 6		2.0 (10, 11)	2.0 (10, Sites 3 and 5; 11, Site 5)^e^
	Area 7		2.0 (9, 12)	0
Clinical experience (years qualified), median (interquartile range), min-max			21.0 (15.5), 5.0-37.0	14.0 (18.0), 5.0-37.0
Delivered groups previously (yes), n (%)			12.0 (92.31)	6.0 (85.7)
**Previous training, n (%)**				
	Communication skills (yes)		7.0 (53.8)	3.0 (42.9)]
	**E-Learning**			
		Yes	2.0 (15.4)	1.0 (14.3)
		No	11.0 (84.6)	6.0 (85.7)
**Preference for training, n (%)**				
	None		1.0 (7.7)	1.0 (14.3)
	Face to face		2.0 (15.4)	1.0 (14.3)
	E-learning and face to face		10.0 (76.9)	5.0 (71.4)

^a^ID: participant identification number.

^b^ID 6 delivered all 6 sessions at Site 2.

^c^IDs 5 and 13 delivered 3 sessions each at Site 4.

^d^ID 1 delivered all 6 sessions at Site 1; ID 3 ceased delivery in Site 6 after session 3.

^e^ID 10 delivered all 6 sessions at Site 3, ID 10 and 11 delivered 3 sessions each at Site 5.

#### Learning

##### Knowledge

Physiotherapists used all nine self-determination theory-based communication strategies in their responses pretraining; the most commonly used strategies were *collaborative goal setting and action planning* and *building relationships*, with no significant change in the rate of use of individual strategies posttraining (Table 2). Knowledge of the SOLAS intervention content and structure improved overall as well as in nine of the 10 intervention components. The use of pain modalities significantly increased posttraining, of which knowledge of content, structure, and group-based exercise programs remained significant following Bonferroni corrections (Table 3).

##### Confidence

Physiotherapists’ confidence significantly increased posttraining overall and for 7 of the 10 individual self-determination theory strategies; *set clear expectations and provide direction* remained significant after Bonferroni correction (Table 2).

Similarly, physiotherapists’ confidence in delivery of the SOLAS content overall and all 10 intervention components significantly increased posttraining; five components remained significant after Bonferroni correction (Table 3).

**Table 2:** Change in physiotherapists’ confidence and knowledge of self-determination theory-based communication strategies.

SDT^a^-based communication strategies	Confidence^b^				Knowledge^c^		
	Median pretraining score (interquartile range), min-max	Median posttraining score (interquartile range), min-max	*Z* score	*P* value	Median pretraining scores (% of physiotherapists)	Median posttraining score (% of physiotherapists)	*P* value
Total	4.5 (1.0), 2.7-5.4	5.0 (0.8), 3.6-5.9	–2.08	.037	8.0 (2.0), 5.0-9.0^d^	7.5 (2.0), 6.0-9.0^d^	.70
Offer a meaningful rationale	5.0 (2.0), 3.0-7.0	7.0 (1.0), 0.0-7.0	–1.80	.07	11.0 (84.6)	8.0 (61.5)	.38
Provide opportunities for patient input and choice	4.0 (3.0), 2.0-7.0	6.0 (1.0), 4.0-7.0	–2.59	.009	6.0 (46.2)	10.0 (76.9)	.22
Use support and encouragement rather than pressurising behaviours	4.0 (2.0), 2.0-6.0	6.0 (2.0), 4.0-7.0	–2.45	.01	3.0 (23.1)	7.0 (53.8)	.29
Set clear expectations and provide direction^e^	5.00 (2.0), 3.0-6.0	6.00 (2.0), 5.0-7.0	–2.85	.004^f^	Not assessed	Not assessed	Not assessed
Collaborative goal setting and action planning^g^	5.0 (3.0), 2.0-7.0	6.0 (1.0), 5.0-7.0	–2.54	.01	12.0 (92.3)	12.0 (92.3)	1.00
Collaborative problem solving	N/A^h^	N/A	N/A	N/A	4.0 (30.8)	8.0 (61.5)	0.13
Provide positive information rich feedback	6.0 (1.0), 4.0-7.0	7.0 (1.0), 4.0-7.0	–1.84	.07	2.0 (15.4)	2.0 (15.4)	1.00
Provide opportunities to practice behaviors	6.0 (2.0), 2.0-6.0	6.0 (1.0), 4.0-7.0	–2.04	.04	3.0 (23.1)	1.0 (7.7)	.63
Acknowledge patients’ feelings and perspectives	6.0 (2.0), 2.0-7.0	7.0 (1.0), 4.0-7.0	–2.09	.04	6.0 (46.2)	9.0 (69.2)	.38
Building relationships	6.0 (1.0), 4.0-7.0	7.0 (1.0), 6.0-7.0	–1.61	.11	12.0 (92.3)	12.0 (92.3)	1.00

^a^SDT: self-determination theory.

^b^Scale range: 1 (not at all good) to 7 (very good).

^c^Percentage of physiotherapists is calculated on the basis of the presence/absence of each SDT strategy in the narrative response.

^d^Values are presented as median (interquartile range), min-max

^e^“Setting clear expectations” was not included in the narrative component of the assessment, as it was not expected to be delivered within the context of the case study.

^f^Significant after Bonferroni adjustment for multiplicity.

^g^Problem solving was considered under the heading of goal setting within the confidence scale.

^h^N/A: not applicable.

**Table 3:** Change in physiotherapists’ confidence and knowledge of the Self-management of Osteoarthritis and Low back pain through Activity and Skills intervention content.

Self-management of Osteoarthritis and Low back pain through Activity and Skills intervention content	Confidence^a^				Knowledge			
	Median pretraining score (interquartile range), min-max	Median posttraining score (interquartile range), min-max	Z score	*P* value	Median pretraining score interquartile range), min-max	Median posttraining score interquartile range), min-max	Z score^b^	*P* value
Total	4.9 (1.8), 3.0-6.0	6.3 (0.8), 4.0-7.0	–3.04	.002^c^	2.0 (2.0), 0.0-6.0	6.0 (1.5), 4.0-7.0	3.09	<.001^c^
Disease mechanisms	5.0 (1.0), 4.0-6.0	7.0 (1.0), 4.0-7.0	–2.49	.01	6.0 (1.0), 4.0-6.0	6.0 (1.0), 5.0-7.0	–2.46	.01
Exercise	6.0 (1.0), 5.0-7.0	7.0 (1), 4.0-7.0	–2.33	.02	6.0 (0.0), 5.0-7.0	7.0 (1.0), 5.0-7.0	–2.82	.005
Physical activity promotion	6.0 (1.0), 2.0-7.0	7.0 (1.0), 4.0-7.0	–2.04	.04	6.0 (1.0), 4.0-7.0	7.0 (1.0), 5.0-7.0	–1.81	.07
Healthy eating and diet	4.0 (2.0), 2.0-6.0	6.0 (1.5), 4.0-7.0	–2.97	.003^c^	4.0 (2.0), 3.0-6.0	6.0 (1.0), 4.0-7.0	–2.72	.006^c^
Relaxation	5.0 (3.0), 1.0-6.0	6.0 (1.5), 4.0-7.0	–2.86	.004^c^	4.0 (3.0), 1.0-7.0	6.0 (1.0), 4.0-7.0	–2.14	.03
Pain-relief techniques	5.0 (2.0), 4.0-6.0	7.0 (1.0), 4.0-7.0	–2.88	.004^c^	6.0 (2.0), 3.0-7.0	6.0 (1.0), 5.0-7.0	–2.48	.01
Medication	4.0 (2.0), 1.0-6.0	6.0 (0.5), 4.0-7.0	–2.88	.004^c^	4.00 (3), 1.0-7.0	6.00 (0.5), 5.0-7.0	–2.73	.006
Pacing	5.0 (2.0), 1.0-7.0	7.0 (1.0), 4.0-7.0	–2.82	.005	6.0 (3.0), 1.0-7.0	6.0 (1.0), 4.0-7.0	–2.50	.01
Mood regulation	4.0 (2.0), 1.0-6.0	6.0 (2.0), 4.0-7.0	–2.83	.005	4.0 (2.0), 1.0-7.0	6.00 (2.0), 4.0-7.0	–2.62	.009
Group-based exercise for osteoarthritis and chronic low-back pain	5.0 (2.0), 2.0-6.0	6.0 (1.0), 4.0-7.0	–3.02	.003^c^	5.0 (1.0), 2.0-6.0	6.0 (1.0), 5.0-7.0	–2.99	.003^c^
Cycle of change^d^	N/A^e^	N/A	N/A	N/A	57.0 (87.7)^f^	61.0 (93.8)^f^	–0.647	.52
Advice to patients^d^	N/A	N/A	N/A	N/A	33.5 (85.9)^f^	36.5 (93.5)^f^	–1.56	.12
Use of pain modalities^d^	N/A	N/A	N/A	N/A	83.0 (79.8)^f^	93.0 (89.4)^f^	–2.33	.02

^a^Scale range: 1 (not at all good) to 7 (very good).

^b^Z score from Wilcoxon signed-rank test.

^c^Significant after Bonferroni adjustment for multiplicity.

^d^Reported as a percentage of the total possible score for the knowledge category.

^e^N/A: not applicable.

^f^Values presented as percentage of the total possible score for the knowledge category and percentage of physiotherapists providing correct responses.

##### Skills

The majority of physiotherapists demonstrated acceptable use of the self-determination theory skill scores during training (median 5.0; interquartile range 1.3; min-max 2.0-6.0), with only two physiotherapists scoring <4.

#### Behavior

Of the six primary care sites that agreed to implement the intervention, five completed delivery and one site ceased delivery after Session 3 due to poor client attendance. Physiotherapists delivered SOLAS to a median of 4.0 (interquartile range 4; range 3-8) participants per class. The total mean %fidelity score (93.5%; SD 4.9%) and the overall fidelity scores were high (≥80%) (Multimedia Appendix 7). The difference between the actual and intended duration of all sessions was not statistically significant, apart from the education component of Session 1, which was significantly longer than the protocol (*P*=.03, Z=–2.23).

Physiotherapists delivered SOLAS in a needs supportive manner consistent with a self-determination theory-based communication style (HCCQ: median 5.2, interquartile range 1.3, min-max 3.7-5.8; CCBS: median 6.6, interquartile range 1.0, min-max 5.6-7.0; Table 4). The SOLAS scale results demonstrated that physiotherapists implemented the intervention overall with acceptable competence (median 4.5, interquartile range 1.2, min-max 2.8-4.8; Table 4). The median scores of only 2 of the 15 self-determination theory strategies were delivered below the competence level during both the education and exercise components of the intervention (ie, *use support and encouragement rather than pressurising behaviors* and *acknowledge patient’s feelings and perspectives*).

### Implementation Outcomes

#### Posttraining Feedback Questionnaire

The median scores for physiotherapists’ ratings of the acceptability, appropriateness, and sustainability of E-SOLAS training to support delivery of the SOLAS intervention were high (Multimedia Appendix 4). All physiotherapists reported that E-SOLAS could be used as a training method in primary care, with 100% of respondents (n=13) recommending it to other primary care physiotherapists and the majority expressing a preference for e-learning alone (69.2%, n=9) over blended learning (30.8%, n=4).

#### Postdelivery Qualitative Interviews

Five of the seven physiotherapists who delivered the SOLAS intervention were interviewed within 2 weeks of program completion. Ten themes were identified from the analysis of participant interview data (Multimedia Appendix 8).

**Table 4:** Physiotherapists’ use of the self-determination theory-based communication strategies during implementation of Session 4 of the Self-management of Osteoarthritis and Low back pain through Activity and Skills.

SDT^a^–based communication strategies^b^			Overall class median score (interquartile range), min-max	Education component median score (interquartile range), min-max	Exercise component median score (interquartile range), min-max	Z score^c^	*P* value
Total			4.5 (1.2), 2.8-4.8	4.3 (1.3), 3.2-5.2	4.5 (2.1), 2.3-5.2	–0.13	.89
**Autonomy support**							
	Offer a meaningful rationale		4.5 (1.8), 3.5-6.5	5.0 (1.5), 4.0-6.0	5.0 (2.5), 3.0-7.0	0	1.00
	Provide opportunities for patient input and choice		4.0 (1.3), 3.5-5.5	4.0 (1.0), 3.0-4.0	5.0 (2.0), 4.0-7.0	–1.84	.07
	Use support and encouragement rather than pressurising behaviours		3.0 (0.8), 2.0-3.0	3.0 (1.0), 3.0-4.0	2.0 (1.0), 1.0-3.0	–1.89	.06
**Structure**							
	Set clear expectations and provide direction		3.0 (3.5), 1.0-6.5	5.0 (2.0), 4.0-6.0	4.0 (4.0), 1.0-6.0	–1.08	.27
	**Goal setting**						
		Review goal setting	3.5 (1.5), 3.0-5.0	5.0 (2.0), 4.0-6.0	3.0 (3.5), 1.0-5.0	–1.62	.10
		Collaborative goal setting	5.0 (2.3), 2.5-5.5	4.0 (2.0), 3.0-6.0	4.0 (3.5), 1.0-6.0	–0.27	.78
		Collaborative action planning	2.5 (2.5), 1.0-5.5	4.0 (4.0), 1.0-5.0	1.0 (4.0), 1.0-6.0	–0.55	.58
		Collaborative barrier identification	4.5 (2.5), 2.5-5.5	4.0 (3.5), 1.0-7.0	5.0 (3.0), 1.0-6.0	–0.13	.89
		Collaborative problem solving	4.5 (2.5), 2.0-5.5	4.0 (3.5), 1.0-7.0	5.0 (3.5), 1.0-6.0	–0.27	.78
	Provide positive encouragement		5.0 (0.8), 3.5-5.0	5.0 (1.5), 3.0-5.0	5.0 (1.0), 4.0-6.0	–1.34	.18
	Provide positive, information-rich feedback		5.5 (2.3), 3.5-6.5	6.0 (2.5), 3.0-6.0	5.0 (2.0), 4.0-7.0	–1.00	.31
	Provide opportunities for patient practice		6.0 (1.0), 4.0-6.0	Not applicable	6.0 (1.0), 4.0-6.0	Not tested	Not tested
**Interpersonal involvement**							
	Acknowledge patients’ feelings and perspectives		2.5 (2.5), 1.0-5.0	3.0 (3.5), 1.0-5.0	1.0 (4.0), 1.0-6.0	–0.18	.85
	**Build relationships**						
		Active listening	5.5 (3.0), 1.5-6.0	6.0 (2.5), 2.0-6.0	5.0 (3.5), 1.0-6.0	–1.73	.08
		Interest in patients	5.5 (1.0), 4.0-6.0	5.0 (1.0), 4.0-6.0	6.0 (1.0), 4.0-6.0	–1.73	.08

^a^SDT: self-determination theory.

^b^Scale range: 1 (not at all good) to 7 (very good).

^c^Z score from Wilcoxon signed-ranks test to assess differences between the education and exercise components across all classes.

#### Acceptability of E-SOLAS

Physiotherapists reported that they had a very positive experience with E-SOLAS training and felt that it was an acceptable and valuable method of training. A number of physiotherapists emphasized the convenience and flexibility of e-learning as a method of training as compared to face-to-face training. The format of training with gate-level assessments and the resource materials contained in E-SOLAS were also viewed positively.

#### Appropriateness of E-SOLAS

All five physiotherapists were positive about the appropriateness of the E-SOLAS content and resources in meeting their practical needs and the needs of their clients in preparing them to deliver SOLAS using needs supportive communication. One physiotherapist also reported gaining greater confidence in managing clients beyond the class setting, whereas another physiotherapist added that the e-learning format had the advantage of allowing her to reflect on learning new skills in relation to the autonomy-supporting style of delivery.

#### Feasibility of E-SOLAS

##### Demand

Although all physiotherapists reported spending additional time reviewing the E-SOLAS content and resources in preparation for delivery of the intervention, they felt the additional time was important for the first delivery of any new program and would reduce with subsequent deliveries (Multimedia Appendix 9).

##### Adaptation

None of the physiotherapists reported deviating from the training specifications; however, all physiotherapists made recommendations for future adaptations to either the E-SOLAS content or training format. These included providing additional resources to guide physiotherapists in educating clients about the health risks associated with the overuse of pain medications, healthy eating guidelines, a wider range of exercise options, and the provision of outcome measures for clinicians to evaluate the intervention independently. Proposed adaptations to the training format included giving participants an estimate of the time required to complete each level and additional e-learning training and blended learning (ie, small-group face-to-face coaching alongside E-SOLAS) to support delivery of the self-determination theory-based communication strategies during goal setting and action-planning activities.

#### Fidelity to E-SOLAS

All physiotherapists aimed to deliver the intervention content and self-determination theory-based needs supportive communication with high fidelity.

#### Sustainability of E-SOLAS

Overall, physiotherapists were positive about the potential for integration of E-SOLAS into existing primary care settings to support the sustained use of the SOLAS intervention as a treatment and reported plans to continue implementation in their service area. One physiotherapist proposed training a designated clinician in each primary care area through face-to-face training, who would act as a peer mentor to support colleagues who completed E-SOLAS to specifically deliver the self-determination theory-based communication strategies.

## Discussion

### Overview

The overall aim of this study was to develop and evaluate an e-learning training program to support physiotherapists to deliver the SOLAS intervention in a primary care setting. The effectiveness of E-SOLAS on physiotherapists’ knowledge, skills, and delivery of the SOLAS intervention was assessed alongside the acceptability and feasibility of the training program. Specifically, results indicated that physiotherapists’ knowledge and confidence increased from pretraining to posttraining assessment and physiotherapists’ behavior was positively influenced by E-SOLAS training, as the SOLAS intervention content and theory-based communication style were delivered with high fidelity. Finally, implementation outcomes posttraining and from the qualitative interviews were overtly positive with regard to the acceptability, appropriateness, feasibility, and potential for future integration of E-SOLAS into existing primary care health services.

### Effectiveness of the E-SOLAS Training Program

Physiotherapists’ confidence in the SOLAS intervention content and self-determination theory-based communication style increased posttraining, which is important because it can indicate how likely a learner (in this case, physiotherapist) is to engage in the required behavior [38,39]. Knowledge also increased for the SOLAS intervention content, but there were limited changes in knowledge of the self-determination theory-based communication strategies. This may be explained by physiotherapists’ high pretraining knowledge levels, suggesting a ceiling effect, as they were highly experienced and the majority had undertaken communication-based training previously, thus limiting their potential for future improvement [40]. These findings mirror our previously published evaluation of SOLAS face-to-face training [15]. The majority of physiotherapists also demonstrated acceptable competence in relation to their skills; however, two physiotherapists were rated below the competence level. Review of the audio recordings revealed that one recording was very short (<2 minutes), and thus, it was difficult to assess it in a meaningful way. Although guidelines were provided on how to conduct the role play, no guidance was given on its duration. Despite the difficulty in prescribing a set amount of time, a minimum time period could have been set to ensure a meaningful assessment, which could be applied for future iterations of E-SOLAS.

In terms of behavior, physiotherapists delivered the intervention as intended, adhering to the intervention content and delivery in a manner consistent with the self-determination theory-based communication style. The mean high fidelity to intervention content based on physiotherapists’ self-reported checklists was 93%, which aligns with the findings of the previous feasibility trial [14]. For assessment of the self-determination theory-based communication style, the physiotherapists’ scores on the two global measures aligned closely with face-to-face training. More specifically, for the HCCQ, the median score was 5.2 in this study and 5.3 (on a 7-point Likert scale) in the face-to-face training study [15]. Scores on the CCBS were consistent for both studies. However, an intervention-specific measure of needs support enables a more focused look at contextual elements [41]. Here, there was some divergence between e-training and face-to-face training, with median scores of 4.5 and 4.0 on a 7-point Likert scale, respectively, favoring E-SOLAS [15].

In this study, two self-determination theory-based communication strategies (*use support and encouragement rather than pressurising behaviours* and *acknowledge patients’ feelings and perspectives*) were delivered with low competence across both the education and exercise components of the class, highlighting the need for further training or adaption to E-SOLAS to further support these strategies. Interestingly, the communication strategies related to goal setting, action planning, and problem solving were delivered to a higher level of competence than the face-to-face training [15]. This may have been due to the additional interactive elements added to E-SOLAS to address the concerns identified by the physiotherapists during the development phase. Furthermore, this improvement in goal setting-related strategies may have inadvertently reduced competence in the communication strategy *use support and encouragement rather than pressurising behaviours*, as emphasis was placed on physiotherapists being more directive with clients regarding goal setting in situations where clients were unable to articulate or formulate a goal themselves. Recent research has highlighted the difficulty in applying effective goal setting in clinical settings [42], and future training programs need to consider these strategies carefully. Overall, E-SOLAS training seems at least as effective as face-to-face training in developing physiotherapists’ knowledge, confidence, and ability to deliver the intervention as intended [22].

### Implementation Outcomes for the E-SOLAS Training Program

Physiotherapists were very positive about E-SOLAS following training and delivery and believed it was an acceptable, appropriate, feasible, and sustainable method of training in primary care. Participants spent a mean of 9 hours completing the training over 16 days while working at their own pace and predominantly in their own time, which has clear advantages over the 12-hour face-to-face training time in addition to travel, cost, and time off work experienced by physiotherapists in our previous feasibility trial. E-SOLAS participants demonstrated high levels of engagement with training, including a 100% completion rate within the specified timeframe. This may reflect the physiotherapist-recognized importance of group-based self-management programs for busy primary care settings as well as the emphasis HCPs now place on a client-centered communication style and the acquisition of behavior-change skills [17]. Furthermore, these high levels of physiotherapists’ satisfaction and engagement could also reflect the systematic and inclusive process used to develop the E-SOLAS training program according to the recommendations of the Medical Research Council [12,43].

In terms of feasibility, technical difficulties can sometimes hamper the success of e-learning with HCPs [44]. Six of the 13 physiotherapists reported difficulty accessing online materials. Therefore, it is important to ensure that technical support is in place to maintain user engagement. One of the main advantages of e-learning is flexibility and control of the time and location for program completion [45], as demonstrated in this study, wherein the majority of participants completed E-SOLAS outside work.

Although physiotherapists were satisfied with the program overall, there were some adaptations suggested, including provision of further information to support the delivery of certain education components, inclusion of details of the estimated time to complete training, and the use of blended learning. These suggestions are in line with the general recommendations for e-learning programs should be tailored to HCPs’ particular knowledge and experience [46]. For example, in the context of E-SOLAS, one physiotherapist may want more information on pain medication, whereas another might like additional videos of communication strategies [45]. Such individualized learning pathways may lead to not only a more engaged learner with enhanced knowledge but also more effective delivery of the intervention.

Despite the high rate of planned implementation of the SOLAS intervention posttraining, the program was fully delivered by six physiotherapists at five sites across four primary care areas. The main reasons for nonimplementation were beyond the scope of the study and were related to the nonavailability of staff. Of the five sites with full implementation, there was an equal mix of sole and shared delivery, in contrast to the previous feasibility trial where all physiotherapists delivered the intervention independently [15]. Physiotherapists who delivered the intervention implemented it with high fidelity, apart from the education component of Session 1, which is consistent with the findings of face-to-face training [14]. Although the qualitative interview findings did not suggest any significant barriers to future implementation by a sole practitioner following training, the suggestion of a local peer mentor and the development of blended learning may be warranted to overcome this potential obstacle.

### Strengths and Limitations

The major strengths of this study are its focus on program development and evaluation within a group of experienced physiotherapists who received e-learning training while working within their primary care setting. Specifically, E-SOLAS was developed and underpinned by theory, with a clear rationale about how the intervention components were developed and adapted. The use of a formal evaluation model [13] allowed for a more comprehensive understanding of the effectiveness of E-SOLAS training, including the objective evaluation of physiotherapists’ behavior during training, which is frequently absent from assessments [23,47,48]. Furthermore, the application of the World Health Organization’s implementation outcomes using mixed methods enabled a comprehensive assessment of the feasibility of implementation of this e-learning training program and required adaptations to increase acceptability [24]. Finally, the assessment of fidelity of intervention delivery using validated measures following e-learning has been rarely reported in the literature and is one of the novel aspects of this study.

A few limitations of this study should be acknowledged. Owing to the relatively small sample size, particularly for the delivery phase of the study, further investigation in a larger sample is warranted. Although a nonvalidated feedback measure was used to evaluate some training and implementation outcomes, its components were informed by a framework for the evaluation of technology-enhanced learning [25] and our face-to-face training feedback measure [15]. Although physiotherapists’ competence to deliver the SOLAS intervention was assessed posttraining, there was no pretraining assessment of their skills, which should be included in future studies [46]. Future studies should also incorporate some form of client measurement to more clearly understand the efficacy of this training approach. Self-report checklists were used to assess the fidelity to intervention content, which is less robust than other methods such as independently rated audio recordings [14]. Any future research evaluating a new program should apply robust fidelity-assessment methods to all parts of the intervention [36,49]. Finally, the role-play activities were an important part of E-SOLAS training; however, they were designed as one-on-one interactions (ie, between the physiotherapist and one client). Therefore, physiotherapists did not get an opportunity to practice their delivery of the intervention in a group setting prior to implementation. Future programs should try to ensure that all elements of the intervention are accurately reflected in the training program.

### Conclusions

The comprehensive evaluation reported in this study provides preliminary evidence of the effectiveness, acceptability, and feasibility of an e-learning program to train physiotherapists to deliver a group-based self-management intervention in a primary care setting that is equivalent to face-to-face training. These findings will inform the development and implementation of a definitive trial and support its scalability to the wider primary care system.

## Appendix

Multimedia Appendix 1 Description of the process used to develop the E-SOLAS program. E-SOLAS: E-learning training program for Self-management of Osteoarthritis and Low back pain through Activity and Skills.

Multimedia Appendix 2 E-SOLAS training program structure and content. E-SOLAS: E-learning training program for Self-management of Osteoarthritis and Low back pain through Activity and Skills.

Multimedia Appendix 3 Outcome measures for evaluation of E-SOLAS training and implementation of the SOLAS intervention. E-SOLAS: E-learning training program for Self-management of Osteoarthritis and Low back pain through Activity and Skills.

Multimedia Appendix 4 Feedback measures used to record physiotherapists' reaction to E-SOLAS training and implementation outcomes posttraining. E-SOLAS: E-learning training program for Self-management of Osteoarthritis and Low back pain through Activity and Skills.

Multimedia Appendix 5 Pre- and posttraining questionnaire for evaluation of physiotherapists' self-reported knowledge and perceived confidence of the SOLAS intervention and self-determination theory-based communication strategies. SOLAS: Self-management of Osteoarthritis and Low back pain through Activity and Skills.

Multimedia Appendix 6 Curatr analytics results.

Multimedia Appendix 7 Fidelity to delivery of SOLAS intervention content. SOLAS: Self-management of Osteoarthritis and Low back pain through Activity and Skills.

Multimedia Appendix 8 Acceptability and appropriateness of E-SOLAS training: Themes and theme examples from participant interviews. E-SOLAS: E-learning training program for Self-management of Osteoarthritis and Low back pain through Activity and Skills.

Multimedia Appendix 9 Feasibility of E-SOLAS training: Themes and theme examples from participant interviews. E-SOLAS: E-learning training program for Self-management of Osteoarthritis and Low back pain through Activity and Skills.

## Abbreviations

CCBS: Controlling Coach Behaviour Scale
E-SOLAS: E-Learning training program for Self-management of Osteoarthritis and Low back pain through Activity and Skills
HCCQ: Health Care Climate Questionnaire
HCP: health care practitioner
ID: participant identification number
SOLAS: Self-management of Osteoarthritis and Low back pain through Activity and Skills
