# Supplementary material for: Evaluation of an E-Learning Training Program to Support Implementation of a Group-Based, Theory-Driven, Self-Management Intervention For Osteoarthritis and Low-Back Pain: Pre-Post Study
Source: J Med Internet Res. 2019 Mar 7;21(3):e11123. doi: 10.2196/11123 (PMC6427104; doi:10.2196/11123)
Supplement: Multimedia Appendix 1 [file jmir_v21i3e11123_app1.pdf]

**Multimedia Appendix 1. Description of the process used to develop the E-SOLAS programme**

The development of E-SOLAS included incorporating several theories of learning and motivation; i.e. social constructivism [27], self-regulated learning theory [28] and SDT [29], and a number of steps.

(1) Focus groups were conducted with primary care PTs who had completed face to face training (n=6) in the SOLAS feasibility trial or were interested in receiving this form of training (n=12) to explore their attitude, knowledge and experience towards e-learning in general and specifically to the SOLAS training programme. Two researchers (AH, HR) independently read the transcripts and used deductive thematic analysis drawing on the Theoretical Domains Framework (TDF) [30] to identify the determinants (i.e., barriers and enablers) of PT engagement with the proposed e-learning training programme ((Table 1A).

(2) Using a combination of theory and evidence, intervention components which could address the above barriers and enablers were chosen by the research team. Specifically, behaviour change techniques were selected from the Behaviour Change Techniques Taxonomy v1[31] to target the identified determinants (Table 1B).

(3) The existing materials from face to face training were adapted for use within an e-learning environment. For example, the brief PowerPoint presentations were edited for e-learning using Articulate Studio '13, a software programme that supports the enhancement of existing PowerPoint presentations with the addition of audio voice-overs, quizzes and other interactions. The existing videos of the exercise components of the intervention and SDT-based communication strategies, and downloadable resources were uploaded to Curatr.

(4) A pilot study was conducted with four PTs who reviewed and provided feedback on the prototype reporting it to be acceptable, relevant and engaging. After minor amendments, the definitive SOLAS E-learning training programme (E-SOLAS) was launched.

**Table 1A Barriers and facilitators to e-learning training and the SOLAS prototype**

| TDF Domain | Barriers | Facilitators |
|---|---|---|
| **Knowledge** | • Computer use in general<br>• Lack of peer discussion and reading text only can limit in-depth understanding | • Provide detailed induction on Curatr<br>• Group-based learning |
| **Skills** | • No opportunity for role play or practice as in face-to-face for immediate feedback<br>• Peer assessment alone insufficient | • Watching video simulation of specific skills of good and poor practice,<br>• Self-reflection and self-appraisal of performance v useful.<br>• Blended learning, opportunity for feedback |
| **Environmental Context & Resources** | • Protected work time to complete the course not supported.<br>• Technical difficulties accessing course from workplace and viewing videos due to poor internet connectivity.<br>• Computer availability at work, Busy and noisy at work.<br>• Lack of familiarisation with Curatr features, i.e. drag and drop, comment boxes, downloading resources | • Do it in own time if CPD points provided,<br>• Detailed induction on technical requirements and Curatr.<br>• Access it from home or on phone – needs 4G.<br>• Flexibility and no travel time |
| **Beliefs about Consequences** | • Peer assessment not sufficient for critical feedback | • Feedback from course facilitator<br>• Clinical relevance, perceived effectiveness and ease of implementation |
| **Beliefs about Capabilities** | • Concern E-learning not sufficient for learning needs supportive communication skills | • Blended learning – opportunity for practice and<br>• Self-reflection in own time and own pace and feedback |
| **Emotion** | • Worry with proposed video-upload of communication skills assessment.<br>• Frustration with technical difficulties of past courses and some prototype aspects | • Use audio recording communication skills<br>• Provide detailed induction and FAQ's to address technical issues |
| **Social Influence** | • Unsure how to engage in online discussion / comments.<br>• Limited networking in online course | • Provide opportunity for interaction with peers<br>• Managerial support for protected learning time during work or study leave.<br>• Positive appraisal from peers about E-learning |
| **Optimism** | • Negative past experience of low quality, boring, text heavy courses | • Well planned blended learning courses with ongoing access |
| **Goals** | • Long courses with no short-term goals. Difficult to motivate self to engage with and complete online courses | • Short-term personal goal at start of training |
| **Reinforcement** | • Lack of incentive | • Willing to complete in own time for CPD points |

**TDF: Theoretical Domains Framework; CPD: Continuing Professional Development**

**Table 1B Description of the process used to develop the E-SOLAS programme**

| Summary of the main barriers and enablers identified from the focus groups | The TDF domains linked to the barriers and enablers identified from the focus groups | BCTs, and specific examples as to how to overcome these barriers and enhance the enablers |
|---|---|---|
| Physiotherapists lacked experience of using e-learning programmes | Knowledge | **BCTs:** Social Support; Demonstration of the behaviour; Behavioural practice/rehearsal<br><br>**Example:** Information from a member of the research team regarding how to use the e-learning platform and what the programme would entail. The provision of written and verbal information on how to use the e-learning platform and how e-learning can support continuous professional development of health care professional |
| Physiotherapists had limited protected time, poor internet connectivity and technical challenges in the work setting. | Environmental context and resources | **BCTs:** Social Support; Restructure the physical environment<br><br>**Example:** Explicit managerial support to complete the E-SOLAS programme; Technical support provided by a member of the research team; Ability to complete the programme outside of the workplace (i.e., at home) provided |
| Physiotherapists were worried about proposed upload of communication skills assessment and frustrated with technical difficulties of past courses | Emotion | **BCTs:** Social Support; Restructure the physical environment<br><br>**Example:** Technical support provided by a member of the research team; Ability to complete the programme outside of the workplace (i.e., at home) provided |
| Physiotherapists were concerned that e-learning would lead to limited opportunities for group discussion, interaction and /or networking | Social influences | **BCT:** Social Support<br><br>**Example**: A virtual discussion group was set up as part of the programme. At various points during the training programme. Physiotherapists had to post and respond to comments in the discussion group. |
| Physiotherapists had concerns about the scope of E-learning to support training in SDT-based communication style of the SOLAS intervention | Beliefs about capabilities, Beliefs about consequences | **BCTs:** Information about the behaviour; Demonstration of the behaviour; Behavioural practice; Self-monitoring; Feedback on the behaviour;<br><br>**Example:** Physiotherapists were provided with written information regarding the use of e-learning to support the training in communication strategies. Additional video–based examples of the SDT-based communication style were also added to the E—SOLAS programme to alleviate physiotherapists' concerns, as were further opportunities to role play |

| | | |
|---|---|---|
| | | and self-reflect on the use of the SDT-based communication style. Finally, greater opportunities for feedback were provided through the E-SOLAS programme, for example, assessments at the end of each level of the E-SOLAS programme |
| Physiotherapists may lack motivation to complete an e-learning programme | Goals | **BCTs:** Goal-setting Material Reward; Prompts & Cues<br><br>Examples: Physiotherapists were encouraged to set a goal at the start of the E-SOLAS training programme, Email reminders were sent to Physiotherapists if they had not started the programme or had not progressed from a certain level by a specified date. A CPD certificate was provided to Physiotherapists on successful completion of the programme within a specified timeframe. |
| Physiotherapists believed the provision of CPD points would act as an incentive | Reinforcement | **BCTs:** Material reward<br><br>**Example:** Physiotherapists were provided with a CPD certificate based on their programme logbook , at the end the course |
| Physiotherapists believed it would opportunities for self-reflection and feedback for skills development | Skills; Beliefs about capabilities | **BCTs:** Behavioural practice; Goal-setting (Behaviour); Action Planning Self-monitoring; Feedback on the behaviour;<br><br>**Example:** Additional opportunities to role play and self-reflect on the use of the SDT-based communication style were added to E-SOLAS programme. In addition, greater opportunities for feedback were provided through the E-SOLAS programme, for example, assessments at the end of each level of the E-SOLAS programme |
| Physiotherapists advised that managerial support would be important | Social influences | **BCTs:** Social Support<br><br>Example: Explicit managerial support to complete the E-SOLAS programme; |

**TDF: Theoretical Domains Framework; BCT: Behaviour Change Technique**
