# Supplementary material for: Evaluation of an E-Learning Training Program to Support Implementation of a Group-Based, Theory-Driven, Self-Management Intervention For Osteoarthritis and Low-Back Pain: Pre-Post Study
Source: J Med Internet Res. 2019 Mar 7;21(3):e11123. doi: 10.2196/11123 (PMC6427104; doi:10.2196/11123)
Supplement: Multimedia Appendix 2 [file jmir_v21i3e11123_app2.pdf]

Multimedia Appendix 2. E-SOLAS training programme structure and content

Level	Level Description	Content of the Level	Sample Materials used during the Level
**Level 1**	An introduction to E-SOLAS programme	1. Welcome by the E-SOLAS research team<br>2. Learning outcomes for the E-SOLAS programme<br>3. Evidence for self-management and the SOLAS intervention<br>4. Overview of the SOLAS programme	• Lectures with audio voiceover<br>• Downloadable training programme handbook<br>• Videos from members of the research team<br>• Group discussion board
	**End of level assessment and feedback**: Multiple Choice Quiz. Pre-set grading by Curatr based on course developers specified answers and feedback automatically provided to the physiotherapists based on the score attained and the answers chosen in the quiz (i.e., a correct or incorrect answer)		
**Level 2**	The SOLAS programme education content for each week of the intervention	1. SOLAS programme – Week 1<br>2. SOLAS programme – Week 2<br>3. SOLAS programme – Week 3<br>4. SOLAS programme – Week 4<br>5. SOLAS programme – Week 5<br>6. SOLAS programme – Week 6	• Lectures with audio voiceover<br>• Downloadable training programme handbook<br>• Copies of the materials required to run each week (e.g. patient activity diary; patient handbook)
	**End of level assessment and feedback**: Short Answer Quiz. Pre-set grading by Curatr based on course developers specified answers and feedback automatically provided to the physiotherapists based on the score attained and the answers chosen in the quiz (i.e., a correct or incorrect answer)		
**Level 3**	An introduction to self-determination theory and the particular communication skills to be used as part of the SOLAS intervention	1. Overview of Self—determination theory<br>2. Developing an awareness of controlling behaviour<br>3. Promoting client autonomy<br>4. Developing client competence – Part 1<br>5. Developing client competence – Part 2<br>6. Building meaningful engagement with clients	• Lectures with audio voiceover<br>• Downloadable training programme handbook<br>• Videos of good and poor practice<br>• Videos of peer role model explaining the use of certain skills<br>• “In-level” activities<br>• Self-reflection exercises
	**End of level assessment and feedback:** Multiple Choice Quiz. Pre-set grading by Curatr based on course developers specified answers and feedback is automatically provided to the physiotherapists based on the answers chosen in the quiz (i.e., a correct or incorrect answer)		
**Level 4**	How to deliver the SOLAS programme: Putting the theory-based communication skills into practice	1. Observation and comment on video interaction between a physiotherapist and client<br>2. Audio recorded role play with a colleague followed by self-reflection<br>3. Audio recorded role play with a	• Lectures with audio voice over<br>• Video of physiotherapist and client interaction<br>• Role-play instruction sheet

		colleague followed by feedback from research team	• Communication skills checklist to be used for self-reflection<br>• Instruction sheet for upload of audio file to Curatr
	**End of level assessment and feedback**: Upload of audio file from role-play for review by a member of the research team. Written tailored feedback is provided via email to each physiotherapist on their use of communication skills. Sample audio-clips are provided to illustrate examples of good and poor practice from the uploaded role-play.		
**Level 5**	The SOLAS programme exercises for each week of the intervention	1. Introduction to the individual exercises<br>2. How to run the exercise component on the exercise component of the SOLAS intervention	• Lectures with audio voice over<br>• Videos of exercises being performed<br>• Copy of the client exercise checklist<br>Copy of exercise pictures and instructions to be used with each exercise
	**End of level assessment and feedback:** Upload of checklist to confirm the exercises which are feasible at the location where the intervention will be run for review by the research team. Written tailored feedback is provided via email to each physiotherapist to confirm the list of exercises provided is appropriate.		
**Level 6**	Conclusion to the E - SOLAS training	1. Concluding remarks by the E-SOLAS research team	• Lectures with audio voice over<br>• Videos from members of the research team
	**End of level assessment and feedback**: Upload of participants' activity log for review by the research team. No feedback was provided to physiotherapists regarding this activity log but the number of training hours recorded by participants' were used to generate a Continuing Professional Development certificate that equated to one point for each hour spent in training.		
