# Supplementary material for: Evaluation of an E-Learning Training Program to Support Implementation of a Group-Based, Theory-Driven, Self-Management Intervention For Osteoarthritis and Low-Back Pain: Pre-Post Study
Source: J Med Internet Res. 2019 Mar 7;21(3):e11123. doi: 10.2196/11123 (PMC6427104; doi:10.2196/11123)
Supplement: Multimedia Appendix 3 [file jmir_v21i3e11123_app3.pdf]

Multimedia Appendix 3. Outcome measures for evaluation of E-SOLAS training and implementation of the SOLAS intervention

Outcomes	Description	Measurement tool	Data Collection Method and Time Point
**Training Outcomes**			
**Participant Reaction**	Participants react favourably to the E-SOLAS training	A feedback measure (18 general questions) related to participant satisfaction, engagement, accessibility and quality of E-SOLAS training overall and the usefulness and relevance of each level (11 level specific questions). **Sample statement:** "The SOLAS Physiotherapist E-learning training programme provided me with useful information and skills that may improve my physiotherapy practice in group settings". ("1-strongly disagree" to "5-strongly agree")	Online survey; Post-training
**Engagement**	Participant interaction with / use of E-SOLAS components and materials.	Curatr analytics for each participant: Number of levels completed; Time spent on training; Number of resources accessed; Number of uploads provided.	Via curatr analytics; Post-training
		Participant activity log (time spent on E-SOLAS and reading resource materials	Upload via Curatr; Post-training
		Feedback measure above (6 questions) **Sample statement:** On average how long did you spend completing training at any one time? (30-60mins; 1-2 hours, > 2 hours)	Online survey; Post-training
**Participant Learning**	Participants acquire intended knowledge, skills, and confidence		
**Knowledge**	Participant self-reported knowledge of the content of SOLAS intervention.	SOLAS intervention structure and content was assessed using a previously validated measure [14] using statements rated on a 7-point Likert scale (from "1-not at all good" to "7-very good"). **Sample statement:** "Please rate your knowledge of providing specific exercise advice to people with osteoarthritis and chronic low back pain" Three narrative questions were also used to establish knowledge of the intervention components using a previously validated measure [15]. Marks were allocated to each question and converted into percentages. **Sample question: "**List three pieces of advice you would give to someone with osteoarthritis/chronic low back pain experiencing a flare-up"	Online survey; Pre & Post-training

	Participant self-reported knowledge of the SDT-based communication strategies	Narrative case study used a previously validated measure [15] in which participants were asked to recall a treatment encounter from the previous week in which they tried to treat and motivate a disengaged client. Responses were coded for the presence or absence of each SDT-based strategy by a blinded rater (AK), with 50% of responses coded by a second expert rater (JM) to ensure accuracy and consistency.	Online survey; Pre & Post-training
**Confidence**	Participant perceived confidence in using the SOLAS intervention content	Confidence in the use of 10 SOLAS intervention content components was assessed using a previously validated measure [15] using a 7-point Likert scale (ranging from "1- not all good" to "7-very good"). **Sample statement:** "Please rate your confidence in providing physical activity prescription to people with osteoarthritis and chronic low back pain"	Online survey; Pre & Post-training
	Participant perceived confidence in using the SDT-based communication strategies	Confidence in the use of 10 SDT-based communication strategies was assessed using a previously validated measure [15] using a 7-point Likert scale (ranging from "1- not all good" to "7-very good"). **Sample statement:** "Please rate your confidence in using autonomy supportive rather than controlling language and behaviour during group based classes".	Online survey; Pre & Post-training
**Skills**	Participant use of SDT-based communication strategies	Role play to practice the use of SDT-based communication strategies following completion of behavioural change components of E-SOLAS (Levels 3 and 4). Role play between PT and colleague acting as a typical or challenging client. PTs requested to apply the SDT-based communication strategies. Role plays were audio-recorded and uploaded for review by a member of the research team with background in health psychology (JM). Each role play was assessed using an eight point checklist and given an overall score on a 7-point Likert scale (ranging from 1-not at all well to "7- very well") for use of the SDT-based strategies.	Upload via Curatr; During training
**Participant Behaviour**			
**Behaviour**	Participant delivery of the SOLAS intervention content as intended.	Participant completion of previously validated checklists for each SOLAS session in which they record the presence/absence/attempt to deliver each intervention component (133 items) [14].	Paper-based checklist; During intervention delivery
	Participant use of the SDT-based communication strategies during the intervention.	Audio recorded delivery of SOLAS session four class per participant, following consent of clients. Each recording was assessed by a blinded rater (with 50% double coded) using three measures:	Audio recording; Post intervention delivery

		▪ 15-item Health Care Climate Questionnaire [34], scored on 7-point Likert scales ranging from 1=strongly disagree'; 7='strongly agree'<br>▪ 5-item version of the Controlling Coach Behaviour Scale [35], scored on 7-point Likert scales ranging from 1=strongly disagree'; 7='strongly agree'<br>▪ Intervention specific measure of needs supportiveness SDT communication strategies; the SOLAS scale [15]. **Sample statement:** For the strategy of 'uses support and encouragement rather than pressurising behaviours'; 1= uses directive, strict, controlling language; 7 = uses supportive, non-controlling language	
**Implementation Outcomes**			
**Acceptability**	Participant perception that E-SOLAS is an agreeable (as credible and acceptable as face-to-face) training option	Feedback measure above. 1-item related to Acceptability was assessed using a 5-point Likert scale (ranging from "1- very unacceptable" to "5-very acceptable")**:** "How acceptable is this E-learning training programme to your health service training needs?<br><br>Semi-structured telephone interview conducted with participants who delivered SOLAS by member of the research team. **Sample question:** "Based on your experience of delivering SOLAS, how acceptable was the E-learning training programme in preparing you to deliver it?	Online survey; Post-training<br><br>Telephone interview; Post intervention delivery
**Appropriateness**	The perceived fit or relevance of the E-SOLAS training for Physiotherapists working in primary care settings.	Feedback measure that included 2-items related to Appropriateness was assessed using 5-point Likert scales (ranging from "1- very irrelevant" to "5-very relevant"). **Sample statement:** "How relevant is this E-learning training programme to your health service training needs?<br><br>Semi-structured telephone interview conducted with participants who delivered SOLAS by member of the research team. **Sample question:** "Based on your experience of delivering SOLAS, how appropriate was the E-learning training programme in preparing you to deliver it?	Online survey; Post-training<br><br>Telephone interview; Post intervention delivery
**Feasibility**	The extent to which E-SOLAS can be used as a training method for Physiotherapists in the primary care settings	Feedback measure that included 2-items related to Feasibility was assessed: **Sample question:** "Overall would you recommend this course to primary care physiotherapists?" Yes; No..If not why not?<br><br>Semi-structured telephone interview conducted with each participant by member of the research team. **Sample question:**	Online survey; Post-training<br><br>Telephone interview; Post

		Do you think this E-learning training alone is sufficient to prepare you to deliver SOLAS or do you think you need additional face-to-face or E-learning training?	intervention delivery
**Fidelity**	The extent to which the SOLAS intervention is implemented as intended in the clinical primary care practice setting	Please review the Behaviour section above in the table.	
		Participant perception of fidelity to SOLAS intervention was assessed in the semi-structured telephone interview conducted with each participant by member of the research team. **Sample question:** How well do you feel you delivered the programme as intended from the training received?	Telephone interview; Post intervention delivery
**Sustainability**	Participants think the E-SOLAS training is sufficient to lead to the sustained use of the SOLAS intervention within the HSE	Feedback measure that included 4-items related to Sustainability was assessed using a 5-point Likert scale (ranging from “1- not very confident” to “5-very confident”). **Sample statement**: “How confident are you that this E-learning training programme is sufficient to lead to the sustained delivery of the SOLAS programme within your HSE primary care area?”	Online survey; Post-training
		Semi-structured telephone interview conducted with each participant by a member of the research team. **Sample question:** “What is your opinion of the feasibility of using the E-learning training programme to support delivery of SOLAS in the HSE primary care settings going forward?	Telephone interview; Post intervention delivery
