# Supplementary material for: Evaluation of an E-Learning Training Program to Support Implementation of a Group-Based, Theory-Driven, Self-Management Intervention For Osteoarthritis and Low-Back Pain: Pre-Post Study
Source: J Med Internet Res. 2019 Mar 7;21(3):e11123. doi: 10.2196/11123 (PMC6427104; doi:10.2196/11123)
Supplement: Multimedia Appendix 4 [file jmir_v21i3e11123_app4.pdf]

Multimedia Appendix 4. Feedback measure used to record Physiotherapists Reaction to E-SOLAS training and Implementation Outcomes post-training

**Satisfaction**	Median [IQR], min-max
1. *How satisfied were you with the SOLAS Physiotherapist e-learning training programme? (*1 'very dissatisfied' to 5 'very satisfied')	5.0 (1.0), 4.0-5.0
2. *The SOLAS Physiotherapist E-Learning training programme was enjoyable* (1 'strongly disagree' to 5 'strongly agree')	4.0 (1.0), 3.0-5.0
**Engagement**	n (%)
3. *Did you find the online programme engaging*? Yes	13.0 (100.0%)
1. *How much time did it take you to complete the full training programme*?	Mean days (SD), min-max
	16.3 (6.03), 9.0-28.0
2. *On average how long did you spend completing the training at any one time*?	n (%)
• 30-60 mins	2.0 (15.4%)
• 1-2 hours	10.0 (76.9%)
• >2 hours	1.0 (7.7%)
3. *How much time did you spend reading downloaded documents whilst completing training*?	
• 30-60 mins	3.0 (23.1%)
• 1-2 hours	5.0 (38.5%)
• 2-5 hours	4.0 (30.8%)
• >5 hours	1.0 (7.7%)
	Median [IQR], min-max
4. *How did you find working independently during training*? (1 'very easy' to 5 'very hard')	1.0 (1.0), 1.0-3.0
5. *Did you find any difficulty not having access to other therapists or a 'teacher'*? (1 'very easy' to 5 'very hard')	1.0 (2.0), 1.0-4.0
**Accessibility of the E-SOLAS training programme and resource materials**	
6. *What proportion of the course did you complete*? (estimate out of 100%)	Mean % (SD), min-max
• At work	23.6 (37.6), 0-100.0
• Outside work in own time	47.9 (44.6), 0-100.0
• Outside work on work time	28.5 (45.1), 0-100.0
	n (%)
7. *Did you encounter any difficulties while undertaking this E-learning training programme*? Yes	9.0 (69.2%)
8. *If yes, please tick as many as relevant*	
• Work time	1.0 (7.7%)

• Home time	3.0 (23.1%)
• Computer access work	4.0 (30.8%)
• Computer access home	2.0 (15.4%)
• Accessing online materials	6.0 (46.2%)
• Completing in-course activities	1.0 (7.7%)
• Completing gate assessments	5.0 (38.5%)
	Median (IQR), min-max

9. *How easy was it for you to* (1 'very easy' to 5 'very hard)

• use the online programme [i.e. accessing the site, logging in]?	2.0 (1.5), 1.0-4.0
• navigate around the site [i.e. getting back to the home page, finding a particular page of interest]?	1.0 (1.0), 1.0-3.0
• access the resource materials?	3.0 (3.0), 1.0-4.0
• find the display/ format of the information to use?	1.0 (1.0), 1.0-4.0

Quality of the E-SOLAS training programme

10. *What did you like about this online training, if anything? (provide examples if you can)?*

11. *What did you dislike about this online training, if anything? (provide examples if you can)?*

12. *How did you find the display/format of the information?* (1 'very poor' to 5 'very good')

• Amount of text per page	4.0 (1.0), 4.0-5.0
• Amount of graphics	4.0 (1.0), 3.0-5.0
• Color of text	5.0 (1.0), 4.0-5.0
• The forum	4.0 (1.0), 3.0-5.0
• Level of interactivity	4.0 (2.0), 2.0-5.0

13. *How would you rate the* (1 'poor' to 5 'excellent')

• quality of presentation of online training programme?	5.0 (1.0), 4.0-5.0
• content of online training programme?	5.0 (1.0), 4.0-5.0
• quality of resource materials within online training programme?	5.0 (1.0), 4.0-5.0

17. How helpful was the UCD team in supporting you during training? (1 'very unhelpful' to 5 'very helpful')	5.0 (0.0), 1.0-5.0
18. *How effectively do you think the content of the course was presented*? (1 'very effective' to 5 'not at all effective')	1.0 (1.0), 1.0-4.0

**Implementation Outcomes**	
**Acceptability of the E-SOLAS training programme**	
19. *How acceptable is this E-learning training programme to your health service training needs?* (1 'very unacceptable' to 5 'very acceptable')	5.0 (0.0), 4.0-5.0
**Appropriateness of the E-SOLAS training programme**	
20. *How relevant is this E-learning training programme to your health service training needs*? (1 'very irrelevant' to 5 'very relevant')	5.0 (0.0), 4.0-5.0
21. *The SOLAS Physiotherapist E-learning -training programme provided me with useful information and skills that may improve my physiotherapy practice in group-based settings*. (1 'strongly disagree' to 5 'strongly agree')	5.0 (1.0), 4.0-5.0
**Feasibility of the E-SOLAS training programme**	n (%)
22. *Overall would you recommend this course to primary care physiotherapists*? Yes	13.0 (100.0%)
23. *Would you have a preference for the method of SOLAS physiotherapist training*?	
• Face to face	0.0 (0.0%)
• Online	9.0 (69.2%)
• Both	4.0 (30.8%)
**Sustainability of the E-SOLAS training programme**	Median (IQR), min-max
24. *How confident are you to deliver the SOLAS programme following this E-learning training programme*? (1 'Not very confident to 5 'Very confident')	4.0 (1.0), 4.0-5.0
25. *The SOLAS Physiotherapist E-Learning Training programme provided me with useful information and skills that will allow me to deliver the SOLAS programme competently*. (1 'Strongly disagree to 5 'Strongly agree')	5.0 (1.0), 4.0-5.0
26. *How confident are you that this E-learning training programme is sufficient to lead to the sustained delivery of the SOLAS programme within your HSE primary care areas*? (1 'Not very confident to 5 'Very confident'),	4.0 (0.0); 4.0-5.0
	n (100%)
27. *Now that you have completed training do you intend to run the SOLAS programme in your service area*? Yes	13.0 (100.0%)

Level specific questions

1. *What is your overall rating of each Level? (1 'poor' to 5 'excellent')*

For all below questions (1 'strongly disagree' to 5 'strongly agree')

2. *This level was sufficiently interactive*

3. *The format of this level was innovative*

4. *The content of this level was interesting*

5. *The content of this level was easy to understand*

6. *The content of this level increased my understanding of the SOLAS programme*

7. *The content of this level increased my confidence in delivering the SOLAS programme*

8. *The resources for this level helped me to understand the training*

9. *The resources for this level were useful*

10. *The level gate assessment was relevant to the content of this level*

11. *The level gate assessment was easy to complete*
