# Supplementary material for: Evaluation of an E-Learning Training Program to Support Implementation of a Group-Based, Theory-Driven, Self-Management Intervention For Osteoarthritis and Low-Back Pain: Pre-Post Study
Source: J Med Internet Res. 2019 Mar 7;21(3):e11123. doi: 10.2196/11123 (PMC6427104; doi:10.2196/11123)
Supplement: Multimedia Appendix 5 [file jmir_v21i3e11123_app5.pdf]

Multimedia Appendix 5. Pre and post-training questionnaire for evaluation of Physiotherapists self-reported knowledge and perceived confidence of SOLAS intervention and SDT-based communication strategies

1. *Please TICK for each question in both sections a) (knowledge) and b) (confidence) in providing physiotherapy management to people with osteoarthritis (OA) and chronic low back pain (CLBP)*

	a) How would you describe your *knowledge* of the following?							b) How would you describe your *confidence* in providing information about and/or using the following to treat people with OA/CLBP?						
	Not at all good		Somewhat good			Very good		Not at all good		Somewhat good			Very good	
Disease mechanisms and causes of OA and CLBP	1	2	3	4	5	6	7	1	2	3	4	5	6	7
Specific exercise advice	1	2	3	4	5	6	7	1	2	3	4	5	6	7
Physical activity prescription	1	2	3	4	5	6	7	1	2	3	4	5	6	7
Balanced diet/healthy eating	1	2	3	4	5	6	7	1	2	3	4	5	6	7
Relaxation techniques	1	2	3	4	5	6	7	1	2	3	4	5	6	7
Pain relieving strategies (ice, heat, TENS)	1	2	3	4	5	6	7	1	2	3	4	5	6	7
Medication (for pain management)	1	2	3	4	5	6	7	1	2	3	4	5	6	7
Pacing (for pain management)	1	2	3	4	5	6	7	1	2	3	4	5	6	7
Anxiety/mood regulation (for pain management)	1	2	3	4	5	6	7	1	2	3	4	5	6	7
Group-based exercise programme for OA and CLBP	1	2	3	4	5	6	7	1	2	3	4	5	6	7

2. How would you describe your knowledge of the SOLAS intervention structure?

Not at all good		Somewhat good			Very good	
**1**	**2**	**3**	**4**	**5**	**6**	**7**

3. How would you describe your knowledge of the SOLAS intervention content?

Not at all good		Somewhat good			Very good	
**1**	**2**	**3**	**4**	**5**	**6**	**7**

4. In your own words, what are the aims of the SOLAS programme?

____

____

5. What are the 5 stages of the cycle of change?

a. ____

b. ____

c. ____

d. ____

e. ____

6. List four types of pain-relieving strategies (and potential cautions for use, if any) that you would recommend to people with OA/CLBP

a. ____**Caution:**_________________________________

b. ____ **Caution:**_________________________________

c. ____ **Caution:** _________________________________

d. ____ **Caution:** _________________________________

7. List three pieces of advice you would give to someone with OA/CLBP experiencing a flare-up:

a. ____

b. ____

c. ____

8. Please CIRCLE for each question, relating to your current delivery of group-based classes

How would you describe your confidence in using the following strategies during group based classes?

	Not at all good		Somewhat good			Very good	
Provide meaningful rationale for the advice or self-management behaviour being recommended to patient(s)	1	2	3	4	5	6	7
Provide opportunity for patient input or choice regarding self-management	1	2	3	4	5	6	7
Use autonomy supportive[a], rather than controlling language and behaviour	1	2	3	4	5	6	7
Provide opportunities for patient initiative & independent work	1	2	3	4	5	6	7
Set clear expectations of the session for patients	1	2	3	4	5	6	7
Engage in collaborative goal setting, action planning, & problem solving with patients	1	2	3	4	5	6	7
Give personalised and positive feedback to patients	1	2	3	4	5	6	7
Provide patients with opportunities to practice behaviours	1	2	3	4	5	6	7
Acknowledge patient' feelings and perspectives	1	2	3	4	5	6	7
Build relationships with patients	1	2	3	4	5	6	7

[a] Autonomy supportive language and behaviour promotes patient empowerment and patients feel they have control over their treatment/plans, whereas controlling language / behaviour is pressurising, directive and guilt inducing
