# Supplementary material for: Evaluation of an E-Learning Training Program to Support Implementation of a Group-Based, Theory-Driven, Self-Management Intervention For Osteoarthritis and Low-Back Pain: Pre-Post Study
Source: J Med Internet Res. 2019 Mar 7;21(3):e11123. doi: 10.2196/11123 (PMC6427104; doi:10.2196/11123)
Supplement: Multimedia Appendix 6 [file jmir_v21i3e11123_app6.pdf]

Multimedia Appendix 6. Curatr Analytics Results

Days spent completing the E-SOLAS training programme	Mean (SD), min-max
	14.6 (5.3), 5.0-22.0
Assessment results from Level gate assessments	Median (IQR), min-max
	81.9% (9.1), 72.5-94.1
Number of times E-SOLAS viewed	Mean (SD), min-max
	51.9 (18.2), 30.0-91.0
	n (%)
Participants completion of E-SOLAS training programme within 4 weeks	13.0 (100.0%)
Participants completion of all 6 Level gate assessments	13.0 (100.0%)
Number of participants who made comments in the group discussion	13.0 (100.0%)
Number of participants who replied to other participants	3.0 (23.1%)
Number of participants who completed all 3 uploads	13.0 (100.0%)
