# Supplementary material for: Evaluation of an E-Learning Training Program to Support Implementation of a Group-Based, Theory-Driven, Self-Management Intervention For Osteoarthritis and Low-Back Pain: Pre-Post Study
Source: J Med Internet Res. 2019 Mar 7;21(3):e11123. doi: 10.2196/11123 (PMC6427104; doi:10.2196/11123)
Supplement: Multimedia Appendix 7 [file jmir_v21i3e11123_app7.pdf]

Multimedia Appendix 7. Fidelity to delivery of SOLAS intervention content

	Mean % (SD)
**Total fidelity score**	93.5% (4.9)
**% mean fidelity score per session** ***(n=number of sessions)***	
Session 1 (n=6)	94.9% (5.8)
Session 2 (n=6)	96.4% (5.1)
Session 3 (n=6)	92.4% (5.5)
Session 4 (n=5)	90.5% (5.8)
Session 5 (n=5)	91.4% (8.5)
Session 6 (n=5)	92.4% (9.9)
**% mean fidelity score per site** ***(n= number of sessions)***	
Site ID 1 (n=6)	97.2% (4.8)
Site ID 2 (n=6)	85.4% (8.1)
Site ID 3 (n=6)	96.2% (5.1)
Site ID 4 (n=6)	93.4% (4.5)
Site ID 5 (n=6)	90.5% (6.2)
Site ID 6 (n=3)	98.7% (2.3)
**% mean fidelity score per category (SD)** ***(n= components)***	
Materials (n=25)	84.0% (12.2)
Introduction and review (n=20)	94.8% (9.1)
Education (n=45)	98.1% (3.0)
Exercise (n=37)	93.4% (8.2)
Review and planning (n=6)	93.8% (12.4)
**Total % mean fidelity score for physiotherapists**	93.8% (4.2)
**% mean fidelity score per physiotherapist** ***(n=sessions delivered)***	
ID 1 (n=6)	96.9% (4.7)
ID 3 (n=3)	98.7% (2.3)
ID 6 (n=6)	85.7% (8.3)
ID 5 (n=3)	93.7% (3.2)
ID 10 (n=9)	96.1% (4.5)
ID 11 (n=3)	92.4% (6.6)
ID 13 (n=3)	92.9% (8.1)
**Class size per session**	Mean (SD)
Session 1 (n=6)	5.7 (2.3)
Session 2 (n=6)	4.8 (2.2)
Session 3 (n=6)	4.8 (2.2)
Session 4 (n=5)	4.8 (2.6)
Session 5 (n=5)	4.2 (3.0)
Session 6 (n=5)	4.8 (2.2)
