# Supplementary material for: Evaluation of an E-Learning Training Program to Support Implementation of a Group-Based, Theory-Driven, Self-Management Intervention For Osteoarthritis and Low-Back Pain: Pre-Post Study
Source: J Med Internet Res. 2019 Mar 7;21(3):e11123. doi: 10.2196/11123 (PMC6427104; doi:10.2196/11123)
Supplement: Multimedia Appendix 8 [file jmir_v21i3e11123_app8.pdf]

Multimedia Appendix 8. Acceptability and Appropriateness of E-SOLAS training -Themes and Theme examples from Participant interviews

Outcome	Definition		
**Acceptability of E-SOLAS training programme**	Defined as the extent to which HSE physiotherapists who have delivered the SOLAS programme consider E-SOLAS training and resource materials as credible and acceptable as a face-to-face training option		
**Themes (n PTs)**	***Sample quotes with participant ID numbers***		
Overall Impressions of E-SOLAS<br><br>*(PTs n=5)*	*'I really like it...I liked the fact that I could learn in my own time. You know I would have done the training in my own time after work. You know when you live in the country you have a lot of travelling to do to keep your CPD up. So that was a big benefit for me and I liked that you can go back over things that you're not sure of. You can take your time over things. You can print it out you know its more user friendly than attending a weekend course.'*. [ID11]	*'The e-learning is brilliant and its great to do stuff online especially when were down the country it is great'* [ID1]<br><br>*'The flexibility I suppose is the synopsis of that you could do it when suits you where suits you for how long suits you. If you only have 20 minutes you've still got 20 minutes of it done... it was easy to navigate and the fact that I did go back to it for some of it you know the fact that we can reference back in to it quite easily'*. [ID6]'.<br><br>**'***im not the most computer loving person so for me e-learning as a general rule wouldn't be the first thing id be gravitating to but saying that I thought the course was well presented on it…'the information on it was very personal the emm the layout the fact that all those resources and things are there at your fingertips to a point did make it useful so I suppose I'd be more keen on doing another e-learning now I suppose after it as opposed to before it'*.. [ID6]	*'I did think it was worthwhile doing…I felt the training was really was very good'*. [ID10]<br><br>*'It was my first time doing an e-learning programme like that and yeah I found it really good. Yeah I really enjoyed the e-learning part of it emm I did it over the four weeks as prescribed em as best I could it might have been even 5 weeks maybe and I quite liked that you could do it in you own time. I did it at home because in work you could concentrate more on it I suppose em at home.. 'Yeah I thought it was just as good as face-to-face training. Yeah I didn't think there were any disadvantages to it…'*. [ID5]<br><br>*'I think what was very helpful was being able to do those quizzes at the end of each stage I do think that was really you know very helpful in terms of really making you take on board you know the information that was provided'*. [ID1]
Overall Impressions of E-SOLAS Support Materials & Resources<br><br>(PTs n=5)	*'The material itself was very straightforward and very well presented…*[ID11]	*'the actual clients handout , that went down really well…I think it was just literally having it they found it actually useful one of the things a couple of them did say is the fact that the exercises*	*'I was really happy with them I thought they were really really helpful and the content was good without being too sort of long winded if you like. Yeah so I think the content was quite*

	'I'm a paper person so I actually have it printed off here in a folder in front of me' [ID1]<br><br>'*I didn't go back online but I did use the physiotherapist booklet, I did look at the client booklet and I did look at all of the actual overheads you know we had got all the information so I suppose I did I had printed off a lot of resources so I did go back through all of the resources absolutely and I felt that it was essential that you were you know all the time sort of referring back to that but I didn't go onto the online stuff that was sort of presented on the slides by Deirdre and James again once I started the programme I had a big folder with all the stuff all the resources down you know written-printed off.'* [ID10]	*are part of that handout so that even though you know you were showing them what to do in the department and that they had practiced to a point you'll often get people going home and going I'm still not sure what I did there or I can't remember so it was a good reminder for them.* [ID6]<br><br>*'I suppose in terms of the resources they were you know I think in general they were excellent'.* [ID10]	*accurate and helpful yeah I thought that was great'.* [ID5]<br><br>*'I would have printed off you know the handbook the client handbook and also the physiotherapist handbook so we would have gone back to that you know every week before the class eh to prepare'.* [ID5]
**Appropriateness of E-SOLAS**	Defined as the perceived fit or relevance of the E-SOLAS training for physiotherapists working in primary care.		
Overall Impressions of Appropriateness of E-learning training for their service needs (PTs n=5)	*I felt it was material that resonated with the clients and made a difference to them and as I say I found them using it outside the programme as well'.* [ID11]<br><br>*'I would feel that*	*'I would have found it you know like it would have been reinforcing a lot of what I had probably had been doing I suppose the real new area would have been around really embracing the sort of self-determination and really em I suppose respecting you know patients*	*'you could reflect on it you know doing it each week I think that actually works quite better especially when you're doing the kind of motivational you're trying to you know motivationally interview people or you're trying to change you're language to have bit of*

	it's very practical I think that definitely it is I think that the e-learning programme is definitely adequate in terms of provision of it in communities'. [ID10]	*autonomy in their know their choice to do or not to do'.* [ID10]<br><br>'*It seems to be a very valuable tool for a physio practice.* [ID11]	*practice or to have the week to work on it I think actually works well'.* [ID5]<br><br>*'It had a good impact on me there and felt it gave me a lot of confidence dealing with my clientele outside the class setting'.* [ID11]
