# Supplementary material for: Evaluation of an E-Learning Training Program to Support Implementation of a Group-Based, Theory-Driven, Self-Management Intervention For Osteoarthritis and Low-Back Pain: Pre-Post Study
Source: J Med Internet Res. 2019 Mar 7;21(3):e11123. doi: 10.2196/11123 (PMC6427104; doi:10.2196/11123)
Supplement: Multimedia Appendix 9 [file jmir_v21i3e11123_app9.pdf]

Multimedia Appendix 9. Feasibility of E-SOLAS training - Themes and Theme examples from Participant interviews

**Feasibility criteria**	**Feasibility Definitions**		
**Demand of E-SOLAS Training**	Defined as the extent to which physiotherapists perceived the demand of participating in the E-SOLAS training programme.		
**Themes (n PTs)**	***Sample quotes with participant ID numbers***		
Time Involved<br><br>*(PTs n=5)*	*'For the first few classes I would have sat down the night before for an hour or so and gone over my materials to get ready for it…I was happy with it yeah. I felt it was worth doing and I feel that in subsequent classes I won't have to do that Ill have more confidence and Ill have a better background… yes I mean the material wouldn't have been new to me it was just to make sure that I was presenting it in a way that followed the SOLAS programme'.* [ID11]	*'Obviously if we go and run it again that will be much less because you know one way or another you're more familiar with It'.* [ID6]<br><br>*'I would say that certainly before every one I would need to be spending a minimum of 30 minutes reviewing the materials for the next day but I would do that in general with any presentation'.* [ID10]<br><br>*'I think it was because it was the first time I was running it but I'm planning on running now again another one in a couple of week and I won't need to spend as much time because I've done it'. [ID1]*	*'I did it in my own time and like that I won't like you know it wouldn't be as long this time it's like giving any new talk or whatever that you know just that you have it clear in your head you feel more confident then in delivering it so yeah I feel I probably use it this time but after this I probably won't need it you know you'll get used to delivering it so I think it is worth it if you're delivering the class on an ongoing basis its good'.* [ID5]
**Adaptation to E-SOLAS training**	The extent to which the E-SOLAS training programme content, resource materials and format will need to be modified to enhance its acceptability for future implementation.		
Suggested changes to E-SOLAS training content<br>*(PTs n=5)*	'*I wondered was it a good idea for us to talk a little bit about the dangers of using a lot of prescription and indeed over the counter*	*'Em no maybe the diet section just a little bit more because again there were more questions sort of coming up with that emm yeah and we don't have a dietician on our primary care team, which is a bit*	'*Emmm not really I suppose the only thing because it was very new to me was the psychological stuff that probably there might have been benefits for me having a little bit more of that?'* [ID10]

	medications…I know from my practice a lot of people on my caseload would be using quite strong medications with quite strong side effects’. [ID11]	*of a disadvantage again because you could maybe refer one or two on if you felt they would benefit but I suppose that’s just a problem with our local service than with the programme you know’.* [ID5]<br><br>*‘…the outcome measures I suppose to introduce those and a satisfaction survey whether you want a generic one as part of the programme or I think a department should be using one anyway’. [ID6]*	*‘because some of the people in our programme progressed so well that we were ready to bring them on further you know bring them on to a different level. So maybe another menu of exercises for progress would be good’.* [ID11]
Suggested changes to E-SOLAS training format *(PTs n=5)*	*‘I think it would have helped a lot if I would have been given an idea of how much time each module would take. I would have em I would have found that helpful in planning the time that I gave to it’.* [ID11]	*‘I think the e-learning was great but I suppose it would have been nice to have do you know just that management of the action plans and just someone correcting your style of how to motivate the people- again just around the motivation and the coaching so nearly even if you had half a day of someone just checking to make sure that you cos There’s a lot of self-critique when it’s the online training and its making yourself aware listening to yourself am I telling this person what to do or am I supporting them on what to do and I suppose just even a half a day of training of someone actually there you doing the little workshop with other people and someone critiquing your style that way probably would be good’.* [ID1]	
**Implementation of SOLAS Intervention**	Factors identified by physiotherapists that may have affected the quality and extent to which the SOLAS intervention was delivered successfully as planned by Physiotherapists who have completed E-SOLAS.		
Views of Fidelity to SOLAS intervention protocol (PTs n=5)	*‘I actually stuck to the programme completely, I didn’t deviate once’.* [ID1]<br><br>*‘Em I suppose you would just like to think it was being delivered in as faithful a way as possible’.* [ID11]	*‘I tried to do it in the format how well I did it im not 100% sure. I think some of it is to try and keep and again I think perhaps it’s my client group is to keep to the exact we’ll say time for the exercise that maybe that we might have had a little bit we’ll say time where they could have ran over a bit because they chat a lot I think more elderly people often can be quite chatty’.* [ID6]	*‘we tried to stick to it as best we could…emm especially cos I suppose it was our first time running it. So we very much stuck to the content in it like the slides in it emm trying to use you know like the autonomous supportive language emm you know trying to encourage them to really make their own goals. Emm you know we really kind of stuck to the training as best we could emm bar like that they went off topic a little bit but we really tried to keep with it to be honest’.* [ID5]
Sustainability of E-SOLAS Training	Physiotherapists views of the suitability of the E-SOLAS training to lead to the sustained use of SOLAS within primary care physiotherapy.		
Positive views about future integration (PTs	*I’d say it’s a very good idea. It’s a way of bringing a well-*	*‘I would say it’s very feasible cos it does suit an awful lot of people’.* [ID6]	*‘Oh I think it’s very feasible yeah yeah absolutely yeah I think it would be very feasible’.* [ID5]

n=5)	*structured and well thought out programme into a more widespread group so I think that it's very feasible'.* [ID11]		
Face-to-face training (PTs n=2)	*'For a larger scale roll out I do think that you need to have someone that's really good coming in and saying ok you're not doing the supportive motivation properly you need to kind of do that or that they would come in and watch one or two of our classes do you know if we were doing it correctly and then do you know you could roll it out then but just have if you just had one or two main people or just one main person in every department just trained up with the face to face stuff that they're very aware of the coaching and the supportive motivation and then everyone else could do the e-learning but then you'd have your main resource person that everyone could go to with questions and things like that'. [ID1]*		*'I suppose face to face you might get things cropping up as they come along you know that people will have points or questions that you can learn from somebody else which again I know you can again see comments on the computer but it's not the same as having a conversation necessarily'. [ID6]*
Physiotherapist plans for future delivery of the SOLAS intervention<br><br>*(PTs n=5)*	*It was such a positive experience that I have no hesitation in saying that I will be delivering another run of it at least one and hopefully further on from that I suppose what I wanted to know as well how do the programme makers feel about me feeding back to my colleagues and some of them delivering the programme?' [ID11]*<br><br>*'I'm planning on running now again another one in a couple of week and I won't need to spend as much time because I've done it because I'll know the first class that's going to take that little bit longer so ill push people along so I think the more you run it the more you become familiar with the material and it will become easier do you know that you wouldn't need to spend as long'. [ID1]*	*'I suppose the ultimate plan is then if its positive as you were saying in whatever for to roll it out'. [ID6]*<br><br>*I already have provisional dates to re-start another programme sort of mid-May…It would probably be a 6 week programme running with a week or 2 of a break and then probably another 6 weeks again. [ID10]*	*'I think where we are we have the same staff for quite a while em in that section and em so would see a lot of the I suppose we wouldn't call them the repeat offenders you every few months they're referred back for the same problem and there I suppose what we would try to do once they've gone through the SOLAS programme now you if they're referred back in we could nearly just ring them and say look we've gone through a lot you know that it is all about self-management and if they wanted to do this programme again perhaps but if not there's not a whole lot else physio we can offer you know…em and perhaps expressing that to GPs as well– and I think that would maybe help with waiting list management with primary care resources which is obviously limited for us all'. [ID5]*
